# Very Low Prostate PET/CT PSMA Uptake May Be Misleading in Staging Radical Prostatectomy Candidates

**DOI:** 10.3390/jpm12030410

**Published:** 2022-03-06

**Authors:** Barak Rosenzweig, Rennen Haramaty, Tima Davidson, Alon Lazarovich, Asaf Shvero, Miki Haifler, Jonathan Gal, Shay Golan, Sagi Shpitzer, Azik Hoffman, Omri Nativ, Yuval Freifeld, Rani Zreik, Zohar A. Dotan

**Affiliations:** 1 Department of Urology, Chaim Sheba Medical Center, Ramat Gan 5211401, Israel; rennen.haramaty@sheba.health.gov.il (R.H.); alon.lazarovich@sheba.health.gov.il (A.L.); asaf.shvero@sheba.health.gov.il (A.S.); zohar.dotan@sheba.health.gov.il (Z.A.D.); 2 Sackler Faculty of Medicine, Tel Aviv University, Tel Aviv 6997801, Israel; tima.davidson@sheba.health.gov.il (T.D.); michael.haifler@sheba.health.gov.il (M.H.); shaygo1@gmail.com (S.G.); 3 Department of Nuclear Medicine, Chaim Sheba Medical Center, Ramat Gan 5211401, Israel; 4 Department of Urology, Shamir Medical Center, Tzrifin 6093000, Israel; jonathan.gal8@gmail.com; 5 Section of Urology, Rabin Medical Center, Petah Tikva 4941492, Israel; sagi.shpitzer@gmail.com; 6 Department of Urology, Rambam Health Center, Haifa 3109601, Israel; azikhof@gmail.com (A.H.); o_nativ@rambam.health.gov.il (O.N.); 7 Ruth and Bruce Rappaport Faculty of Medicine, Technion Israel Institute of Technology, Haifa 3200003, Israel; yuvalfrei@gmail.com (Y.F.); ranizreik@gmail.com (R.Z.); 8 Department of Urology, Carmel Medical Center, Haifa 3436212, Israel

**Keywords:** prostate-specific membrane antigen (PSMA), imaging, prostate cancer, radical prostatectomy, staging

## Abstract

Purpose: to evaluate a unique subpopulation of radical prostatectomy (RP) candidates with “negative” prostate ^68^Ga-labeled prostate-specific membrane antigen (PSMA) positron emission tomography (PET) computed tomography (CT) imaging scans and to characterize the clinical implications of misleading findings. Materials and Methods: This case-control retrospective study compared the final histological outcomes of patients with “negative” pre-RP PSMA PET/CT prostate scans (with a prostate maximal standardized uptake value [SUVmax] below the physiologic uptake) to those with an “intense” prostatic tracer uptake (with a SUVmax above the physiologic uptake). The patients underwent an RP between March 2015 and July 2019 in five academic centers. Data on the demographics, comorbidities, prostate-specific antigen (PSA) and rectal exam findings, prior biopsies, imaging results, biopsies, and RP histology results were collected. Results: Ninety-seven of the 392 patients who underwent an RP had PSMA PET/CT imaging preoperatively. Fifty-two (54%) had a “negative” uptake (in the study group), and 45 (46%) had a “positive” uptake (in the control group). Only the lesion size and SUVmax values on the PSMA PET/CT differed between the groups preoperatively. On the histological analysis, only the ISUP score, seminal vesicles invasion, T stage, and positive margin rates differed between the groups (*p* < 0.05), while 50 (96%) study group patients harbored clinically significant disease (ISUP ≥ 2), with an extra-prostatic disease in 24 (46%), perineural invasion in 35 (67%), and positive lymph nodes in 4 (8%). Conclusions: Disease aggressiveness generally correlated with an intense PSMA uptake on the preoperative PSMA PET/CT, but a subpopulation of patients with clinically significant cancer and aggressive characteristics showed a deceptively weak PSMA uptake. These data raise a concern about the unqualified application of PSMA PET/CT for staging RP candidates.

## 1. Introduction

The accurate staging of prostate cancer patients is a crucial step in therapeutic management, sometimes discriminating between a curable and non-curable disease. ^68^Ga-labeled prostate-specific membrane antigen (PSMA) positron emission tomography (PET)-computed tomography (CT) imaging plays an important role in the staging of disease and the evaluation of its recurrence [1,2,3,4]. PSMA PET/CT was recently suggested as a suitable replacement for conventional imaging by providing superior accuracy compared to the combined findings of CT and bone scanning for staging patients with high-risk prostate cancer before a curative-intent treatment. That alternative was considered an important landmark that may affect future guideline recommendations [5].

While the PSMA is generally weakly expressed in normal prostate tissue, it is strongly upregulated in prostate cancer. A PSMA PET/CT sensitivity is correlated with the Gleason score, reaching as high as 84% for a Gleason score > 4 [6]. However, despite the high-level sensitivity of PSMA PET/CT findings, it seems that some clinically significant prostate cancers lack an intense PSMA expression. Silver et al. reported extracorporeal samples of two of thirty-five primary prostate adenocarcinomas, one of eight lymph node metastases, and ten of eighteen prostate tumors metastatic to the bone that did not display tumor cell PSMA immunostaining [6], and Frumer et al. described findings suggestive of its limited role in the clinical setting [7]. In the current work, we evaluated a unique subpopulation of prostate cancer patients with “negative” prostate PSMA PET/CT scans, focusing upon the clinical implications of such potentially misleading results.

## 2. Materials and Methods

Following Helsinki approval and waiver of informed consent, we retrospectively reviewed the medical records of patients who underwent radical prostatectomy (RP) for clinically localized prostate adenocarcinoma in 5 medical centers between March 2015 and July 2019. Only patients who underwent PSMA PET/CT imaging before surgery were included. We collected data on demographics, comorbidities, prostate-specific antigen (PSA) levels, rectal exam findings, magnetic resonance imaging (MRI), PSMA PET/CT results, and histology of former and current biopsies, as well as RP specimens.

The PSMA PET/CT scans were performed in the 5 participating medical centers on integrated PET/CT scanners 50–60 min after injection of 3–5 mCi ^68^Ga–PSMA, as previously described by our group [7]. Prior to the PET/CT study, an intravenous iodine contrast medium was administered to all patients, except to those with known iodine hypersensitivity or renal insufficiency. Diluted iodinated contrast material (800–1000 mL) was administered orally for bowel opacification. Contrast-enhanced multidetector CT was performed from the skull base to the mid-thigh. The acquisition time was 2 min per bed position. The imaging studies were interpreted by experienced specialists in nuclear medicine and radiology. Ga 68-PSMA activity was quantified by calculating a maximum standardized uptake value (SUVmax) by manually generating a region of interest over the sites of abnormally increased radioactive material activity. We defined a group of patients with “negative” PSMA PET/CT prostate scans as those whose prostate SUVmax was below physiologic uptake of 6.6 (the study group) [8]. Patients with PSMA PET/CT scans with prostate SUVmax levels above physiologic uptake comprised the control group [8]. Patients treated with androgen deprivation therapy prior to undergoing PSMA PET/CT imaging were excluded from the analysis.

The RP was performed by means of either the DaVinci© robotic system (Intuitive Surgical Inc., Mountain View, CA, USA), (*n* = 76, 78.3%) or by open surgery (*n* = 21, 21.6%). The RP histology specimens were processed by routine pathologic fixation with formalin solution and evaluated by dedicated uropathologists blinded to preoperative imaging findings. The clinically significant disease was defined as International Society of Urological Pathology (ISUP) ≥ 2. Baseline characteristics and imaging and histology results were compared between the 2 groups.

## 3. Statistical Analysis

The Chi-squared and Mann–Whitney tests were applied for all analyses (IBM SPSS Statistics for Windows, Version 26.0. Armonk, NY, USA: IBM Corp) and a 2-sided *p*-value of <0.05 was taken as significant.

## 4. Results

In total, the medical charts of 392 patients were reviewed, of whom 97 underwent preoperative PSMA PET/CT imaging. Fifty-two (54%) patients fulfilled the study inclusion criteria and formed the study group, and the remaining 45 (46%) comprised the control group. The patients’ median age was 66 years (interquartile range [IQR], 62–70), and there was no significant difference in the baseline comorbidities between the groups (Table 1). The preoperative median PSA level was 8.5 (IQR 6.3–13). The prostate size and the finding of a suspected prostate lesion per digital rectal examination did not significantly differ between the study groups (Table 2).

The preoperative MRI identified 76 suspected lesions defined as Prostate Imaging–Reporting and Data System (PI–RADS) ≥ 3, and there was no significant group difference in the number of lesions graded PI–RADS ≥ 3 (*p* = 0.269, Table 2). While lesion size did not significantly differ between the study and control groups on the preoperative MRIs, the median lesion size on the preoperative PSMA PET/CT was significantly different: it was 11.5 mm (IQR 10–20) vs. 23 mm (17–27), respectively (*p* = 0.015).

A wide range of cutoffs was proposed to detect significant prostate cancer, ranging from the SUVmax 3.15 to up to the SUVmax 9.1 [9,10,11,12]. In the current work, we applied the SUVmax of 6.6 cutoff, as previously described by Uprimny et al. [8]. As dictated by the study design, the median lesion SUVmax in the study group was significantly lower compared to the SUVmax of the control group (3.9 [IQR 3.2–5] and 11.5 [IQR 7.2–16.4], respectively, *p* < 0.001). None of the study group patients harbored a clinically insignificant disease according to the preoperative histology results, and positive lymph nodes were found in four (8%) of their preoperative PSMA PET/CT imaging studies (Table 3).

The final prostatectomy pathology report stated that the average prostate size was 56.8 cc ± 27.95 for the study group and 48.3 cc ± 26 for the control group (*p* = 0.083). The control group had higher T stage levels (*p* = 0.016) and ISUP grades (*p* = 0.009), as well as higher rates of seminal vesicle invasion (*p* = 0.006) (Table 3). Ten (19.2%) study group patients and 22 (48.9%) controls had positive margins (*p* = 0.002) (Table 3). The examination of other characteristics, such as the extra-prostatic extension and perineural invasion, failed to reveal any significant group differences (Table 3). A lymph node dissection was performed in 37 study patients (71.1%) and 35 controls (77.8%), and it identified similar rates of a positive lymph node (stage N1) disease for both groups (Table 3). Finally, the preoperative PET PSMA identified two of the four study patients and two of the three controls as having an N1 stage disease.

## 5. Discussion

PSMA PET/CT has exhibited superior accuracy than the combined findings of CT and bone scanning in staging patients with high-risk prostate cancer before a curative-intent treatment [5]. The PSMA is generally weakly expressed in normal prostate tissues and strongly upregulated on PSMA PET/CT in prostate cancer specimens. There are, however, some important exceptions of clinically significant malignancies in which the uptake is deceptively low. We evaluated this unique subpopulation of prostate cancer patients with “negative” prostate PSMA PET/CT scans, and our findings advocate caveats to the unrestricted adoption of this innovative imaging technique. ^68^Ga-PSMA PET/CT detects the location and extent of primary prostate cancer [13] and is sensitive to PSA kinetics [14]. Its superiority over conventional imaging modalities has been reported for the staging and detection of recurrent disease [14]. Moreover, it adds important information to standard CT/MRI studies, changing treatment strategies in a significant number of patients [15]. Indeed, Gleason scores and PSA levels have been correlated with ^68^Ga-PSMA-11 PET/CT tracer accumulation in primary tumors. For example, Uprimny et al. described that prostate cancer patients with lower PSA values and Gleason scores of six and seven showed a significantly lower ^68^Ga-PSMA-11 uptake [8]. In addition to the primary tumor evaluation on ^68^Ga-PSMA-11 PET/CT images, the tracer accumulation was suggested as being able to identify malignant nodal involvement, representing another crucial step in preoperative staging and evaluation [16,17]. While these data support the application of this modality in primary tumor staging, contradicting evidence limits the enthusiasm over its immediate clinical acceptance. For example, Frumer et al. identified some limitations of ^68^Ga-PSMA-11 PET/CT in identifying lymph node involvement on the preoperative evaluation [7].

Our current data generally reflected a correlation between the prostate cancer histology and the ^68^Ga-PSMA-11 PET/CT uptake; however, they also identified a subpopulation of patients with clinically significant prostate cancer that demonstrated only weak tracer uptake in the index prostatic lesion. The fact that our “negative” PSMA group included patients with significant cancer, and even high-risk characteristics, may point to a possible caveat to its unqualified application. Furthermore, finding a similar percentage of the N1 disease among the “negative” PSMA group patients, while their preoperative PET PSMA identified a similar, if not an even lower, rate of positive nodes, supports the presence of limitations of this imaging approach in staging candidates for radical prostatectomies. An example of a prostate cancer patient subpopulation that lacks a strong PSMA PET/CT tracer uptake includes men with neuroendocrine histology, for which PSMA-targeted imaging was described as being ineffectual [18]. In addition, patients with advanced castration-resistant metastatic disease, especially after failing several lines of chemotherapy, exhibited some sites of disease and lost PSMA expression [19]. These data suggest that some forms of aggressive/resistant traits lack a PSMA uptake. The fact that PSMA functions as a folate hydrolase and that it is expressed in a range of normal tissues and other benign and malignant processes suggests “other end of the spectrum” false scans scenarios [19]. Prostate cancer heterogeneity, described both in the genomic and phenotypic/clinical level, may suggest a possible mechanism explaining this imaging’s lacunae [20,21,22]. While tracer-guided imaging is a well-accepted staging method in oncology, similar to our findings, caveats were advised for its application in many other cancers, such as lung [23,24] and thyroid [25]. We, therefore, suggest that prior to broadly adopting this imaging technique, it must be borne in mind that a small proportion of prostate carcinomas exhibit no or only minimal uptake on PSMA PET/CT.

Blood sugar levels have been correlated with a PET–*fluorodeoxyglucose* (FDG) uptake, suggesting the need to follow special protocols in order to prepare diabetic patients for undergoing PET–FDG imaging [26,27]. Although seen here only as a trend, our results suggest a correlation between the diagnosis of diabetes and a lower prostate SUV uptake on PSMA PET/CT (*p* = 0.07, Table 1). That finding is intriguing given that a PSMA PET/CT tracer does not include fluorodeoxyglucose. One possible explanation may be that prostate cancer patients with diabetes may represent a unique subpopulation in which the PSMA expression correlates with both the androgen receptor expression as well as insulin and the insulin growth factor receptor expression [28].

We recognize that our study has several limitations in addition to its retrospective nature. Our having gathered data from five medical institutes subjects it to interobserver variability, including the radiology and histology analyses. The rarity of applying PSMA PET/CT imaging for staging prostatectomy candidates, however, necessitated such a team effort in order to assemble a large enough cohort of patients who complied with our study inclusion criteria. The fact that radiologists and pathologists in all of the participating medical centers are highly experienced and dedicated professionals may partially compensate for this effect.

Looking forward, we hope that the introduction of new tracers, such as the ^18^F-PSMA-1007, as well as the combination of PSMA PET/CT with MRI, may further contribute to future precision in staging localized prostate cancer [29,30,31,32], possibly overcoming the aforementioned caveat in applying PSMA PET/CT-guided imaging. Until this goal is realized, we believe that the current application of PSMA PET/CT should be carried out in full awareness of possible pitfalls in the unqualified staging of RP candidates.

## 6. Conclusions

Prostate cancer aggressiveness generally correlates with an intense uptake of the PSMA on preoperative PSMA PET/CT; however, there is a subpopulation of patients with clinically significant cancer and aggressive characteristics that show only weak PSMA uptake. These data raise concern over the unqualified application of PSMA PET/CT for the staging of RP candidates.

## Figures and Tables

**Table 1:** Patient characteristics.

Characteristic	Study Group (*n* = 52)	Control Group (*n* = 45)	All Patients (*n* = 97)	*p*-Value
Age (years) (mean +/− SD) Comorbidities (%)	65.8 ± 5.58	65.27 ± 5.54	65.54 ± 5.54	0.646
IHD	10 (19.2)	9 (20)	19 (19.6)	0.88
DM	18 (34.6)	8 (17.8)	26 (26.8)	0.07
HTN	32 (61.5)	23 (51.1)	55 (56.7)	0.30
BMI	28.67 ± 4.2	28.4 ± 4.76	28.52 ± 4.46	0.625

SD, standard deviation; IHD, ischemic heart disease; DM, diabetes mellitus; HTN, hypertension; BMI, body mass index.

**Table 2:** Preoperative patient characteristics.

	Study Group (*n* = 52)	Control Group (*n* = 45)	All Patients (*n* = 97)	*p*-Value
Preoperative PSA (ng/mL) (median, IQR)	7.82 (5.73–11.18)	9.25 (6.5–16.93)	8.5 (6.3–13)	0.059
Suspicious prostate on DRE (%)	27 (51.9)	31 (68.9)	58 (59.8)	0.215
Estimated prostate size (cc) (median, IQR) (per DRE)	40 (30–50)	30 (30002D40)	36 (30-40)	0.12
No. of suspected lesions on preoperative MRI (PIRADS ≥ 3) (median, IQR)	1.39 ± 0.7	1.6 ± 0.8	1.52 ± 0.7	0.269
Lesion size on MRI (mm)	10 (6.5-21)	15.5 (13.25–19)	14 (8–19)	0.227
Lesion size on PSMA PET/CT (mm)	11.5 (10–20)	23 (17–27)	16 (11–23)	0.015
Lesion SUV max (median, IQR)	3.9 (3.2-5)	11.5 (7.2–16.4)	5.15 (3.63–9.2)	<0.001
ISUP preoperatively				
ISUP 1	0	1 (2.2)	1 (1)	0.175
ISUP 2	21 (40.4)	15 (33.3)	36 (37.1)	
ISUP 3	15 (28.8)	8 (17.8)	23 (23.7)	
ISUP 4	7 (13.5)	15 (33.3)	22 (22.7)	
ISUP 5	5 (9.6)	4 (8.9)	9 (9.3)	
NA	4 (7.7)	2 (4.4)	6 (6.2)	
Suspected lymph nodes per PSMA PET/CT (%)	4 (7.7)	7 (15.6)	11 (11.3)	0.223

PSA, prostate-specific antigen; IQR, interquartile range; DRE, digital rectal exam; PIRADS, prostate imaging reporting and data system; MRI, magnetic resonance imaging; SUV, standard value uptake; ISUP, International Society of Urological Pathology; PSMA PET/CT, ^68^Ga-labeled prostate-specific membrane antigen (PSMA) positron emission tomography (PET) computed tomography (CT) imaging.

**Table 3:** Pathologic characteristics per group, per prostatectomy histology. Staging is defined per National Comprehensive Cancer Network (NCCN).

	Study Group (*n* = 52)	Control Group (*n* = 45)	All Patients (*n* = 97)	*p*-Value
Prostate size on pathology (gr) (mean ± SD)	56.8 ± 27.95	48.3 ± 26		0.083
Pathology T Stage (%)				0.016
T2x	17 (32.7)	8 (17.8)	25 (25.7)	
T2a	1 (1.9)	0	1 (1)	
T2b	0	1 (2.2)	1 (1)	
T2c	10 (19.2)	8 (17.8)	18 (18.5)	
T3x	2 (3.8)	0	2 (2)	
T3a	20 (38.5)	19 (42.2)	39 (40.2)	
T3b	2 (3.8)	9 (20)	11 (11.3)	
Pathology N1 stage (%)	4 (7.7)	3 (6.7)	7 (7.2)	1
Pathology ISUP (%)				0.009
ISUP 1	1 (1.9)	0	1 (1)	
ISUP 2	28 (53.8)	17 (37.8)	45 (46.4)	
ISUP 3	19 (36.5)	17 (37.8)	36 (37.1)	
ISUP 4	1 (1.9)	2 (4.4)	3 (3.1)	
ISUP 5	2 (3.8)	8 (17.8)	10 (10.3)	
NA *	1 (1.9)	1 (2.2)	2 (2)	
EPE (%)	24 (46.2)	27 (60)	51 (52.7)	0.173
SVI (%)	2 (3.8)	10 (22.2)	12 (12.4)	0.006
PNI (%)	35 (67.3)	38 (84.4)	73 (75.2)	0.213
Positive margins (%)	10 (19.2)	22 (48.9)	32 (32.9) 0.04 (0.004–0.11)	0.002
Postoperative PSA (ng/mL) (IQR)	0.04 (0.0–0.07)	0.04 (0.0–0.17)		0.617

* The prostatectomy histology was not available for 2 patients whose preoperative histology was ISUP2. T2x-2 pathologic stage, not farther defined; ISUP, International Society of Urological Pathology; EPE, extra-prostatic extension; SVI, seminal vesicle invasion; PNI, perineural invasion; PSA, prostate-specific antigen; stdev, standard deviation; and IQR, interquartile range.

## Data Availability

The data presented in this study are available on request from the corresponding author. The data are not publicly available due to confidentiality of medical records.

## References

[1] Perera M., Papa N., Christidis D., Wetherell D., Hofman M.S., Murphy D.G., Bolton D., Lawrentschuk N. (2016). Sensitivity, Specificity, and Predictors of Positive 68 Ga–Prostate-specific Membrane Antigen Positron Emission Tomography in Advanced Prostate Cancer: A Systematic Review and Meta-analysis. Eur. Urol..

[2] Rauscher I., Düwel C., Haller B., Rischpler C., Heck M.M., Gschwend J.E., Schwaiger M., Maurer T., Eiber M. (2018). Efficacy, Predictive Factors, and Prediction Nomograms for ^68^Ga-labeled Prostate-specific Membrane Antigen–ligand Positron-emission Tomography/Computed Tomography in Early Biochemical Recurrent Prostate Cancer after Radical Prostatectomy. Eur. Urol..

[3] Corfield J., Perera M., Bolton D. (2018). ^68^Ga-prostate specific membrane antigen (PSMA) positron emission tomography (PET) for primary staging of high-risk prostate cancer: A systematic review. World J. Urol..

[4] Morawitz J., Kirchner J., Lakes J. (2021). PSMA PET/CT vs. CT alone in newly diagnosed biochemical recurrence of prostate cancer after radical prostatectomy: Comparison of detection rates and therapeutic implications. Eur. J. Radiol..

[5] Hofman M.S., Lawrentschuk N., Francis R.J. (2020). Prostate-specific membrane antigen PET-CT in patients with high-risk prostate cancer before curative-intent surgery or radiotherapy (proPSMA): A prospective, randomised, multicentre study. Lancet.

[6] Silver D.A., Pellicer I., Fair W.R. (1997). Prostate-specific membrane antigen expression in normal and malignant human tissues. Clin. Cancer Res..

[7] Frumer M., Milk N., Rinott M.G. (2020). A comparison between ^68^Ga-labeled prostate-specific membrane antigen-PET/CT and multiparametric MRI for excluding regional metastases prior to radical prostatectomy. Abdom. Radiol..

[8] Uprimny C., Kroiss A.S., Decristoforo C. (2017). ^68^Ga-PSMA-11 PET/CT in primary staging of prostate cancer: PSA and Gleason score predict the intensity of tracer accumulation in the primary tumour. Eur. J. Nucl. Med. Mol. Imaging.

[9] Rüschoff J.H., Ferraro D.A., Muehlematter U.J. (2021). What’s behind ^68^Ga-PSMA-11 uptake in primary prostate cancer PET? Investigation of histopathological parameters and immunohistochemical PSMA expression patterns. Eur. J. Nucl. Med. Mol. Imaging.

[10] Woythal N., Arsenic R., Kempkensteffen C. (2018). Immunohistochemical Validation of PSMA Expression Measured by ^68^Ga-PSMA PET/CT in Primary Prostate Cancer. J. Nucl. Med..

[11] Demirci E., Kabasakal L., Şahin O.E. (2019). Can SUVmax values of Ga-68-PSMA PET/CT scan predict the clinically significant prostate cancer?. Nucl. Med. Commun..

[12] Zhou C., Tang Y., Deng Z. (2022). Comparison of ^68^Ga-PSMA PET/CT and multiparametric MRI for the detection of low- and intermediate-risk prostate cancer. EJNMMI Res..

[13] Fendler W.P., Schmidt D.F., Wenter V. (2016). ^68^Ga-PSMA PET/CT detects the location and extent of primary prostate cancer. J. Nucl. Med..

[14] Giovacchini G., Giovannini E., Riondato M. (2017). PET/CT with ^68^Ga-PSMA in Prostate Cancer: Radiopharmaceutical Background and Clinical Implications. Curr. Radiopharm..

[15] Grubmüller B., Baltzer P., D’Andrea D., Korn S., Haug A.R., Hacker M., Grubmüller K.H., Goldner G.M., Wadsak W., Pfaff S. (2018). ^68^Ga-PSMA 11 ligand PET imaging in patients with biochemical recurrence after radical prostatectomy—diagnostic performance and impact on therapeutic decision-making. Eur. J. Nucl. Med. Mol. Imaging.

[16] Herlemann A., Wenter V., Kretschmer A., Thierfelder K.M., Bartenstein P., Faber C., Gildehaus F.J., Stief C.G., Gratzke C., Fendler W.P. (2016). ^68^Ga-PSMA Positron Emission Tomography/Computed Tomography Provides Accurate Staging of Lymph Node Regions Prior to Lymph Node Dissection in Patients with Prostate Cancer. Eur. Urol..

[17] van Leeuwen P.J., Emmett L., Ho B., Delprado W., Ting F., Nguyen Q., Stricker P.D. (2017). Prospective evaluation of 68Gallium-prostate-specific membrane antigen positron emission tomography/computed tomography for preoperative lymph node staging in prostate cancer. BJU Int..

[18] Bakht M.K., Derecichei I., Li Y., Ferraiuolo R.M., Dunning M., Oh S.W., Hussein A., Youn H., Stringer K.F., Jeong C.W. (2018). Neuroendocrine differentiation of prostate cancer leads to PSMA suppression. Endocr. Relat. Cancer.

[19] Hofman M.S., Hicks R.J., Maurer T. (2018). Prostate-specific membrane antigen PET: Clinical utility in prostate cancer, normal patterns, pearls, and pitfalls. Radiographics.

[20] Haffner M.C., Zwart W., Roudier M.P. (2021). Genomic and phenotypic heterogeneity in prostate cancer. Nat. Rev. Urol..

[21] Beauregard J.M., Blouin A.C., Fradet V. (2015). FDG-PET/CT for pre-operative staging and prognostic stratification of patients with high-grade prostate cancer at biopsy. Cancer Imaging.

[22] Paschalis A., Sheehan B., Riisnaes R. (2019). Prostate-specific Membrane Antigen Heterogeneity and DNA Repair Defects in Prostate Cancer. Eur. Urol..

[23] Cheran S.K., Nielsen N.D., Patz E.F. (2004). False Negative Findings for Primary Lung Tumors on FDG Positron Emission Tomography: Staging and Prognostic Implications. Am. J. Roentgenol..

[24] Jiang C., Han Y., Hu X. (2014). Relative low glycometabolism may also occur in invasive lung adenocarcinoma with visceral pleural invasion: Case report and comments. Medicine.

[25] Long N.M., Smith C.S. (2011). Causes and imaging features of false positives and false negatives on 18F-PET/CT in oncologic imaging. Insights Imaging.

[26] Rabkin Z., Israel O., Keidar Z. (2010). Do hyperglycemia and diabetes affect the incidence of false-negative 18F-FDG PET/CT studies in patients evaluated for infection or inflammation and cancer? A comparative analysis. J. Nucl. Med..

[27] Pattison D.A., MacFarlane L.L., Callahan J. (2019). Personalised insulin calculator enables safe and effective correction of hyperglycaemia prior to FDG PET/CT. EJNMMI Res..

[28] Lutz S.Z., Hennenlotter J., Scharpf M.O. (2018). Androgen receptor overexpression in prostate cancer in type 2 diabetes. Mol. Metab..

[29] Privé B.M., Israël B., Schilham M.G.M. (2020). Evaluating F-18-PSMA-1007-PET in primary prostate cancer and comparing it to multi-parametric MRI and histopathology. Prostate Cancer Prostatic Dis..

[30] Hong J., Liu B., Wang Z.Q. (2020). The value of 18F-PSMA-1007 PET/CT in identifying non-metastatic high-risk prostate cancer. EJNMMI Res..

[31] Giesel F.L., Knorr K., Spohn F. (2019). Detection efficacy of 18 F-PSMA-1007 PET/CT in 251 patients with biochemical recurrence of prostate cancer after radical prostatectomy. J. Nucl. Med..

[32] Grubmuller B., Baltzer P., Hartenbach S. (2018). PSMA ligand PET/MRI for primary prostate cancer: Staging performance and clinical impact. Clin. Cancer Res..

